# The AML-associated K313 mutation enhances C/EBPα activity by leading to C/EBPα overexpression

**DOI:** 10.1038/s41419-021-03948-6

**Published:** 2021-07-05

**Authors:** Ian Edward Gentle, Isabel Moelter, Mohamed Tarek Badr, Konstanze Döhner, Michael Lübbert, Georg Häcker

**Affiliations:** 1 Institute of Medical Microbiology and Hygiene, Medical Center - University of Freiburg, Faculty of Medicine, 79104 Freiburg, Germany; 2 Department of Internal Medicine III, University Hospital of Ulm, Ulm, Germany; 3 Division of Hematology, Oncology and Stem Cell Transplantation, University of Freiburg Medical Center, Faculty of Medicine, Hugstetter Str. 55, 79106 Freiburg, Germany; 4 BIOSS Centre for Biological Signalling Studies, University of Freiburg, 79104 Freiburg, Germany

**Keywords:** Acute myeloid leukaemia, Cell biology

## Abstract

Mutations in the transcription factor C/EBPα are found in ~10% of all acute myeloid leukaemia (AML) cases but the contribution of these mutations to leukemogenesis is incompletely understood. We here use a mouse model of granulocyte progenitors expressing conditionally active HoxB8 to assess the cell biological and molecular activity of C/EBPα-mutations associated with human AML. Both N-terminal truncation and C-terminal AML-associated mutations of C/EBPα substantially altered differentiation of progenitors into mature neutrophils in cell culture. Closer analysis of the C/EBPα-K313-duplication showed expansion and prolonged survival of mutant C/EBPα-expressing granulocytes following adoptive transfer into mice. C/EBPα-protein containing the K313-mutation further showed strongly enhanced transcriptional activity compared with the wild-type protein at certain promoters. Analysis of differentially regulated genes in cells overexpressing C/EBPα-K313 indicates a strong correlation with genes regulated by C/EBPα. Analysis of transcription factor enrichment in the differentially regulated genes indicated a strong reliance of SPI1/PU.1, suggesting that despite reduced DNA binding, C/EBPα-K313 is active in regulating target gene expression and acts largely through a network of other transcription factors. Strikingly, the K313 mutation caused strongly elevated expression of C/EBPα-protein, which could also be seen in primary K313 mutated AML blasts, explaining the enhanced C/EBPα activity in K313-expressing cells.

## Introduction

Acute myeloid leukaemia (AML) is characterised by the accumulation of granulocyte or monocyte precursors in the bone marrow and peripheral blood. Genomic alterations in AML effect numerous transcription factor genes [1, 2]. In ~10% of AML cases, mutations in the gene encoding the transcription factor C/EBPα are found [3, 4].

The C/EBP proteins are basic region leucine zipper (bZIP), dimeric transcription factors. The most notable haematopoietic defect of C/EBPα-deficient mice is the lack of mature granulocytes [5]. C/EBPα is required for commitment to the granulocytic lineage and in early granulopoiesis, but probably not at later stages [6]. Overexpression of C/EBPα forces the differentiation of bi-potential progenitor cells along the granulocytic lineage [7]. C/EBPα^−/−^ mice do not develop myeloid leukaemia even upon expression of Bcr-abl [8], and C/EBPα is required for the development of leukaemia in other models (MLL-fusion or Hoxa9/Meis1-induced leukaemia) [9, 10].

The C/EBPα gene contains no introns. Two protein isoforms (p42 and p30) are translated by two alternative start codons, with p30 lacking the N-terminal 117 amino acids [11]. The p42 isoform contains an N-terminal transactivation domain and a C-terminal bZIP domain [11]. p30 and p42 appear to have non-redundant molecular functions as shown by their differing influence on AML progression as well as differences in target gene induction [12]. In about two-thirds of the AML cases with C/EBPα-mutations, biallelic mutations are found. N-terminal mutations disrupt the translation of p42 while permitting translation of the p30 isoform, and appear to have some dominant-negative activity. C-terminal mutations generate proteins deficient in DNA binding and/or homodimerization [3, 13–15].

Mice expressing only p30 develop AML-like disease from committed myeloid progenitors [16, 17]. C-terminal C/EBPα-mutations cause expansion of pre-malignant haematopoietic stem cells and a block of granulocytic differentiation [17]. However, the C/EBPα-dependent molecular and signalling changes that lead to malignant transformation are still in many ways unclear [6, 18]. For instance, both types of mutated proteins (N-terminal truncation (p30) and a C-terminal (aa 304–323) duplication) have no activity on a C/EBPα-promoter when transfected into 293 T cells [19], whereas an earlier study found that p30 has substantial albeit reduced activity [20]. Moreover, an N-terminal truncation, but not an AML-associated C-terminal mutation, was found to have a suppressive (dominant negative) effect on wt C/EBPα transcriptional activity in 293 T cells, whereas the opposite effect was observed on the expression of G-CSFR (a target of C/EBPα) in mouse myeloid cells [19]. Different cellular systems and experimental approaches may account for such differences. C/EBPα is known to cooperate with a number of additional transcription factors and chromatin-modulating enzymes [21], and the cellular background is, therefore, likely to be important.

We here used an experimental system of granulocyte differentiation to study molecular effects of C/EBPα-mutations. This model uses committed neutrophil progenitor cells expanded from the bone marrow of mice by the expression of oestrogen-controlled Hoxb8 in the presence of stem cell factor (SCF) [22]. Importantly, their differentiation is very close to that of primary cells and includes the neutrophil propensity to undergo spontaneous apoptosis [23]. Using this system, we expressed C/EBPα-mutations that are associated with human AML, focussing in particular on the K313-duplication. Expression of C/EBPα-K313 repressed neutrophil differentiation. We show that the K313 mutation remains functional in driving gene expression but leads to significant overexpression of both isoforms of C/EBPα.

## Methods

### Cell culture

Hoxb8 neutrophil progenitors were cultured in progenitor outgrowth medium (Optimem Glutamax supplemented with 10% foetal bovine serum (FCS), 1% Pen/Strep, 30 µM β-mercaptoethanol, 1% SCF supernatant, 1 µM β-estradiol). Cells were grown at 37 °C, 5% CO_2_.

### Generation of Hoxb8 neutrophil progenitor cells

HoxB8 cells were generated as previously described [22]. In brief, bone marrow cells from C57Bl/6 mice were stimulated in Roswell Park Memorial Institute supplemented with recombinant mouse cytokines IL-3 (10 ng/ml), IL-6 (20 ng/ml), and 1% SCF supernatant from SCF secreting CHO cells and cultured for 3 days. Cells were infected with retrovirus expressing ER-Hoxb8 fusion, which is active in the presence of estradiol and grown in media containing β-estradiol for 2 weeks to ensure the outgrowth of transformed progenitors. Hoxb8 positive neutrophil progenitors were differentiated by washing out β-estradiol 2–3× with warm phosphate-buffered saline (PBS). To differentiate, cells were seeded at 1 × 10^5^cells/ml in 3 ml differentiation medium (Optimem Glutamax, 10% FCS, 1% Pen/Strep, 1% SCF supernatant) in a six-well plate and cultured for 4 days.

To generate C/EBPα-overexpressing cells, HoxB8 progenitors were infected with pMIG retroviruses expressing either wild type or the indicated mutants of C/EBPα. As pMIG contains an IRES-GFP, cells were sorted for GFP+ populations to select for infected cells. For each construct, multiple infections were performed at different times to avoid clonal selection.

### Luciferase reporter assay

Hoxb8 neutrophil progenitor cells were transduced with the lentiviral construct pF3XC/EBP-Luc, which was made by PCR amplifying the 3X×C/EBP-Luc cassette from pGL3-C/EBP-Luc (a kind gift from Hans Häcker- University of Utah) and cloning it into the pF5XUAS-SV40_PURO [24] vector after cutting with PacI and BamHI (Primers: Fwd- gcctTTAATTAAATTGCGCAATTTGATATCGGA, Rev- gtggGGATCCTTATCGATTTTACCACATT). Cells were selected with 3 µg/ml puromycin. On the day of analysis, cells were counted, washed and 2 × 10^5^ cells were seeded in 150 μL into 96-well plates in duplicate. In all, 30 µL d-luciferin (100 µg/ml) were added, incubated for 10 min (37 °C, 5% CO_2_) and luminescence measured for 10 s using the Tecan infinite M200 reader.

### Inhibition of translation

Hoxb8 neutrophil progenitors or day 1 differentiated neutrophils were seeded at 1 × 10^6^ cells/ml in 3 ml of progenitor or differentiation medium. Cycloheximide was added at 10 µg/ml for indicated times and cells harvested and lysed in 100 µL Laemmli buffer at 95 °C for 10min.

### Cell death staining

In all, 200 µL of cell suspension were taken out of the culture well and mixed with 1 µg/mL propidium iodide and analysed by Flow cytometry. Propidium iodide-positive cells were considered dead cells.

### Determination of proliferation of Hoxb8 neutrophil progenitors

In all, 1 × 10^5^ cells were seeded in 3 ml of progenitor outgrowth medium into six-well plates. Cells were counted using the Casy Cell Counter each day and re-seeded every second day to provide optimal growth conditions.

### Testing for capacity to form colonies and colony size

HoxB8 progenitors expressing the indicated constructs were plated at 50,000 cells per plate in 3.5 cm culture dishes containing OPTIMEM-methylcellulose media containing β-estradiol to prevent differentiation. Cells were cultured for 7 days. Colonies were counted on a Keyence BZ-9000 microscope and sizes of the colonies were measured as the area of pixels for each colony.

### RNA extraction, cDNA synthesis, and quantitative real-time PCR for C/EBPα levels

Total RNA was isolated from 4 × 10^6^ Hoxb8 neutrophil progenitor or differentiated cells using Trizol-Chloroform extraction followed by purification using the High Pure RNA isolation kit (Roche). cDNA synthesis was performed using reverse transcription using Transcript First-strand cDNA synthesis kit (Roche).

Relative mRNA expression was determined either using the Light Cycler Taqman Master kit (Universal Probe Library system, Roche, #67 for mouse C/EBPα and Universal Probe Library system #64 for mouse β-Actin). Expression of the target gene was normalised to β-actin. C/EBPα as well as other mRNAs were also quantified using SYBR green master mix (Thermo) on a Quantstudio (Thermo) machine with 386-well plates with the indicated primers. Fold changes in gene expression were calculated using the ΔΔct method with actin as an internal standard.

### qPCR analysis of human primary AML blasts

All AML BM samples were enriched for mononuclear cells by Ficoll, which normally results in the enrichment of blasts >80%. In all, 1 × 10^7^ cells were lysed using Trizol reagent as recommended in manufacturer’s instructions. Trizol extracted samples were then separated into mRNA, DNA and protein fractions as described in manufacturer’s instructions. mRNA was then transcribed into cDNA using the first-strand cDNA synthesis kit (Roche). qCPR was done using SYBR green master mix (Thermo) on a Quantstudio machine with 386-well plates with the indicated primers. Fold changes in gene expression were calculated using the ΔΔct method with actin as an internal standard.

### qPCR primers

**Table:** 

Gene	Species	Primer sequence 5’−3’
C/EBPα	Mouse	Fwd: AAACAACGCAACGTGGAGA Rev: GCGGTCATTGTCACTGGTC
C/EBPα	Human	Fwd: TATAGGCTGGGCTTCCCCTT Rev: AGCTTTCTGGTGTGACTCGG
S100A9	Mouse	Fwd: TTAGCCTTGAAGAGCAAGAAGATG Rev: AAGGTGTCGATGATGGTGGTT
ID2	Mouse	Fwd: TGTCCTTGCAGGCATCTGAA Rev: ATGCCATTTATTTAGCCACAGAGT
CD14	Mouse	Fwd: CAGAGAACACCACCGCTGTA Rev: CACGCTCCATGGTCGGTAG
CCL3	Mouse	Fwd: TACAGCCGGAAGATTCCACG Rev: TCAGGAAAATGACACCTGGCT
Actin	Human	Fwd: CCTGGCACCCAGCACAAT Rev: GCCGATCCACACGGAGTACT
Actin	Mouse	Fwd: CTAAGGCCAACCGTGAAAAG Rev: ACCAGAGGCATACAGGGACA
SFI3*	Mouse	Fwd: CACTTGGAGCTGGCCTGAG Rev: TGGCATTTCTACTCCAGAGCTTC
PTGS2*	Mouse	Fwd: CTCATTTGCGTGGGTAAAAGC Rev: CCTCTCTGCTTCAGTGAGTTG
IL6Ra*	Mouse	Fwd: GGTGCTGAAGCTCCTCTTGG Rev: CGCCATCCTACTGGGCTTTC
C/EBPα	Mouse	Fwd: CCACTCACCGCCTTGGAAAG Rev: TGCCTGCTGGGTCTTAGAGC
C/EBP Luciferase reporter*		Fwd: GGACAGCAGAGATCCAGTTTGGTTAA Rev: AGCCCGAATTCATCGATGATATCAGA

* Denotes primers used for CHIP qPCR.

### Staining of cell surface markers

Cells were incubated with Fc block (BD – cat. No. 553142), washed stained in PBS 0.5% BSA. Analysis was done using BD FACS Canto II (BD Biosciences). Cells were stained for CD11b (BD Biosciences Cat. no. 561689), Gr-1(BD Biosciences Cat. no. 553129), c-kit (e-biosciences, cat. no. 17–1172), CD14 (Biolegend Cat. no. 150106) and CD115 (e-bioscience Cat. no. 12-1152-82).

### Giemsa staining

Cells were transferred onto microscope slides using a Cytospin Cytocentrifuge, fixed with methanol for 5 min and incubated in Giemsa staining solution.

### Lysate preparation, western blotting

Lysates were prepared by lysing 3 × 10^6^ cells in 100 µL Laemmli buffer at 95 °C for 10 min. Protein concentration was determined by DC Protein Assay (BioRad) and 50 µg of protein was loaded onto poly-acrylamide gels. For human AML samples, the protein fraction recovered from trizol extraction was isolated using the manufacturer’s instructions. Approximately equal loading was made based on loading controls.

### Determination of IL-6 and TNF levels by ELISA

Hoxb8 neutrophil progenitor and d4-differentiated cells were seeded at 1 × 10^6^ cells/ml in 12-well plates. Cells were stimulated with 1 µg/ml LPS for 8 h. Supernatants were used for enzyme-linked immunosorbent assay (E-Biosciences TNF - Cat. no. 88-7324-88, IL-6 - Cat. no. 88-7064-88).

### Adoptive transfer of Hoxb8 neutrophil progenitor cells

In all, 5 × 10^6^ Hoxb8 progenitor cells carrying pMIG-R1, pMIG-FLAG-C/EBPα-wt, or pMIG-FLAG-C/EBPα-K313 together with 0.5 × 10^6^ unfractionated bone marrow cells (C57Bl/6) were injected into the tail-veins of 6–8 week old female C57Bl/6 lethally irradiated (2 × 5.5 Gy) mice. Mice were bled or killed at indicated times. Hoxb8-derived cells were identified as GFP-positive. All animal experiments were performed according to ethical guidelines and were approved by the regierungspresiudium Freiburg (G13-009).

### Microarray analysis of Day 1 differentiated HoxB8 progenitors expressing wild-type or K313 mutated C/EBPα

Microarray data sets and descriptions of experimental conditions can be found at (https://www.ncbi.nlm.nih.gov/geo/query/acc.cgi?acc=GSE120473). To identify overrepresented biological terms most significant DEGs exceeding FDR of <0.001 and Fold change of two were considered for downstream analysis. Overrepresented Gene ontologies including BP, CC, and MF were identified using the R package clusterProfiler [25] (Carlson, 2009) using the compareCluster and enrichGO functions, and visualised using the R packages ggplot2 and patchwork [26] (https://cran.r-project.org/web/packages/patchwork/index.html). Pathways with an adjusted *p* value < 0.05 (Benjamini–Hochberg) were considered significant.

### FLAG-C/EBPα CHIP

FLAG-C/EBPα CHIP was performed based loosely on a previously published protocol [27]. In all, 1 × 10^7^ HoxB8 progenitor cells expressing either empty vector, wild-type C/EBPα or K313 C/EBPα were harvested, washed 2× in warm PBS and then fixed for 10 min in 1% formalin. For C/EBP luciferase reporter CHIP, cells infected with the reporter construct were used. Cells were then rinsed in glycine (125 mM in PBS) for 10 min before being further washed in PBS (10 mL) two times. Cells were resuspended in 200 µl of lysis buffer (40 mM Tris.Cl (pH 8.0), 300 mM NaCl, 1% Triton X-100, 4 mM EDTA (pH 8.0), 1× Complete protease inhibitor cocktail (Roche) and 1 mM PMSF) and incubated on ice for 20 min. Cells were then sonicated using a Biorupter (Biorupter Plus) in separate 100 µL aliquots using Bioruptor tubes (Diagenode #C30010013) on high for 60–70 cycles of 30 s on 30 s off. This was done in 10 cycle batches after which samples were shaken back to the bottom of tubes before continuing. DNA shearing was analysed by agarose 1% gels. Lysates representing ~5 × 10^6^ cells were then diluted in CHIP dilution buffer (40 mM Tris.Cl pH 8.0, 4 mM EDTA (pH 8.0)) incubated with 5 µg of anti-FLAG M2 antibody (Sigma # F1804) for 1.5 h at 4 °C. Antibodies were then precipitated using Dynabead protein G magnetic beads (ThermoFisher). Beads were washed five times with 1 mL of wash buffer (10 mM Tris.Cl (pH 8.0), 150 mM NaCl, 0.1% (vol/vol) Triton X-100) with incubations of 1–2 mins for each wash. Beads were then resuspended in 200 µl TE buffer with 200 mM NaCl. Cross-links were reversed by incubation overnight at 65 °C with shaking. Samples were then treated with 50 µg/mL proteinase K at 45 °C for 1 h before RNAse treatment and purification of DNA using the Qiagen genomic DNEASY blood and tissue prep kit (Qiagen). qPCR was performed using Sybr green master mix (Roche) using the indicated primers, which were previously published [28] (supplemental methods). As a negative control for the CHIP, PCR was done using Sfi3 for normalisation of input amounts. Empty vector controls served as negative controls for the FLAG antibody. All results were then normalised to the Sfi3 values and then to the empty vector controls for calculating fold enrichment.

### Transcription factor enrichment analysis

Gene lists generated from the microarray of C/EBPα-wild type and C/EBPα-K313 were analysed using the CHEA3 webserver [29]. The top 10 enriched transcription factors based on the mean rank score were taken and network analysis done within the CHEA3 tool. As a comparison with endogenously regulated C/EBPα target genes, the differentially regulated genes from [30] (GSE61468) were analysed in the same way.

### CHIP-Seq analysis

CHIP-Seq data from [31] was analysed using EASeq software [32] as follows. Regions were identified by peak finding of either C/EBPα-wild type or C/EBPα-K313 as the sample using Input as a negative control using adaptive local thresholds. The procedure does with some modifications resemble that of MACS [33]. The size of the DNA fragments was set to 150 bp for all samples. Each data set was divided into 100 bp windows, and the reads within each window were scanned genome-widely. A normalisation coefficient (NCIS) serving to normalise the background levels of the two data sets was analysed in accordance with Liang and Keles [34]. As an exception from the NCIS procedure, the window size was entered manually and set to 100 bp. Global thresholds were calculated based on a Poisson Distribution using the genome-wide average number of reads in the windows and an entered *p* value of 1E-05. Adaptive thresholds were modelled the following way: the average number of negative control reads in areas corresponding to 10×, 5× and 250× window size (100 bp) was calculated for each position. This number was used as lambdas for Possion distributions and thresholds that matched an entered *p* value of 1E-05 were calculated. The most conservative threshold was chosen from the three local thresholds of the control and the global threshold of the sample. Thresholds from the negative control were scaled according to the NCIS normalisation factor. The Position and statistics of windows passing the most conservative threshold, and having an NCIS-normalised log2-fold Sample/Control-ratio above 2, as well as <3:1 difference between the signal on the plus and minus strands were extracted into a separate list. This analysis was repeated four times—each time the windows were shifted 25 bp. Windows within 100 bp of each other and overlapping windows were merged. For each region in the resulting list, the borders were refined by sliding a window of 100 bp from one window-size upstream to downstream of the temporary border. The exact position where the number of sample reads within the window fell below the threshold was defined as new border of that region. Shoulders were excluded at values below µ +2 SD. After border refinement and peak-merging, peaks were positively selected for an FDR value of 1E-05 or better and a minimum NCIS-normalised log2-fold difference of 2. All peaks were mapped to the mm9 mouse genome assembly.

## Results

### Expression of AML-associated C/EBPα mutations alters differentiation of Hoxb8 cells

**Fig. 1:**
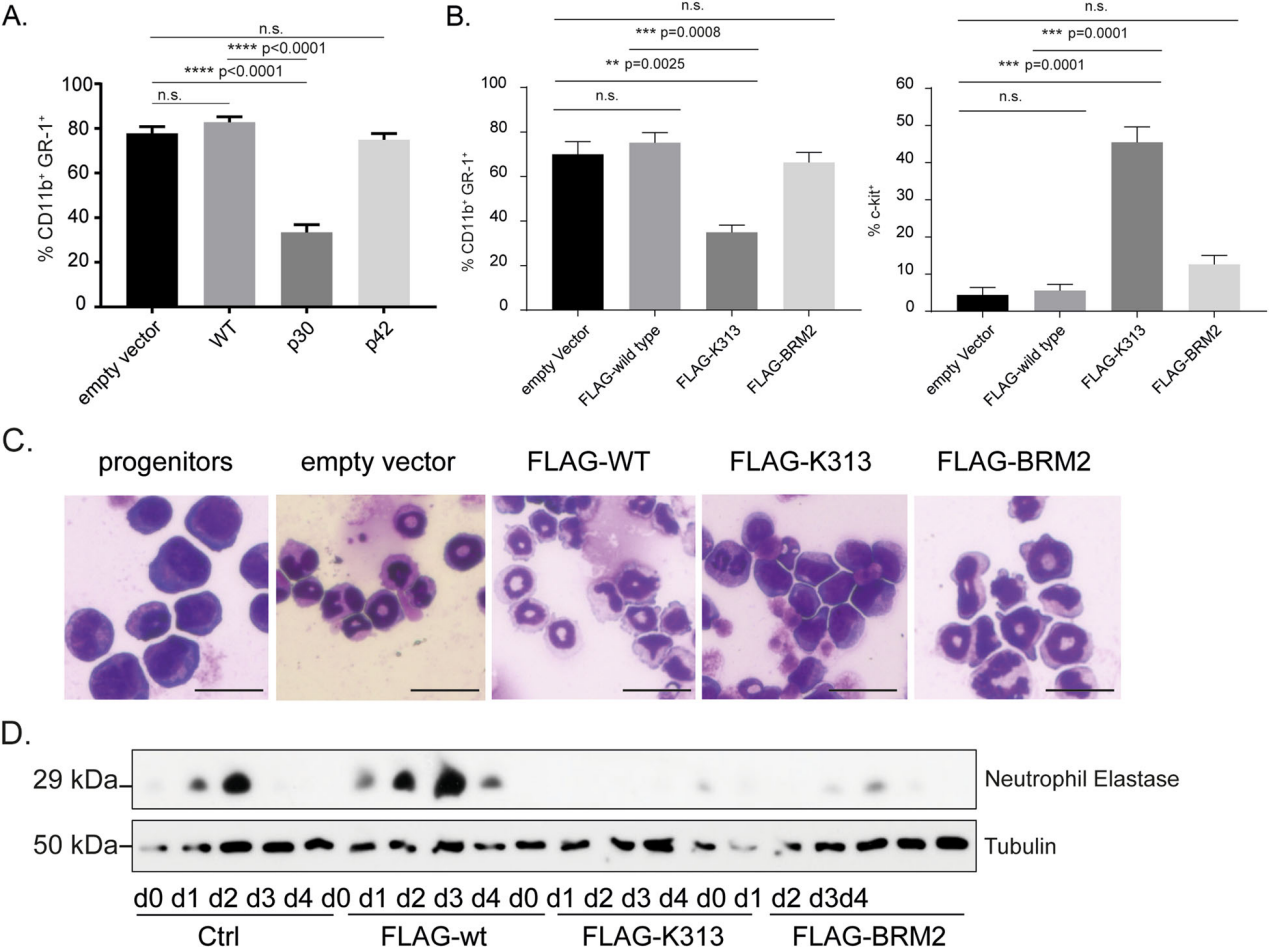
Overexpression of mutant C/EBPα can block differentiation of HoxB8-immortalised neutrophil progenitors in vitro. **A** Quantification of Gr-1/CD11b double-positive populations during differentiation of wt progenitor cells expressing untagged C/EBPα−wt, C/EBPα-p30, or C/EBPα-p42. Quantifications show mean and SEM of at least three independent experiments. *P* values were calculated using one-way ANOVA with multiple comparisons. **B** Quantification of Gr-1/CD11b double-positive and of c-Kit positive populations during differentiation of wt progenitor cell expressing FLAG-tagged C/EBPα−wt, C/EBPα-K313, C/EBPα-BRM2. Progenitor cells were differentiated for 4 days, stained for Gr-1 and CD11b or c-kit. Quantifications show mean and SEM of at least three independent experiments. *P* values were calculated using one-way ANOVA with multiple comparisons. **C** Giemsa stain of day 4 differentiated HoxB8 neutrophils expressing indicated C/EBPα constructs. Scale bars 25 µM. **D** HoxB8 neutrophil progenitors from cells expressing the indicated C/EBPα constructs were differentiated for the indicated number of days and analysed by western blot for neutrophil elastase.

Confirming differentiation of Hoxb8 neutrophils, when Hoxb8 was removed by withdrawing oestrogen, progenitors differentiated over 4 d into a population of cells mostly expressing CD11b, Gr-1 and low levels of c-kit (Fig. S1A). These cells are very similar to slightly immature neutrophils. We first tested the effect of retroviral expression of wt (both isoforms), p42 or p30 C/EBPα (Fig. S1B) in progenitor cells. Expression of wt and p42 C/EBPα had no obvious effect on differentiation of the cells. p30-expression strongly reduced Gr-1 expression, suggesting a dominant-negative effect (Figs. 1A, S1C). We then tested two typical AML-associated C/EBPα C-terminal mutations, the duplication of the lysine K313 (K314 in mouse, here referred to as K313) and the BRM2 mutation (I294 and R297 in humans, I295A and R298A in mouse).

Expression of untagged or C-terminally FLAG-tagged C/EBPα-K313 both had a strong differentiation-blocking effect, by cell surface staining (CD11b, Gr-1, c-kit, Fig. 1B, Fig. S1D, E) and morphology (Fig. 1C); BRM2 had only a very mild effect on surface marker expression and the cells were somewhat larger but otherwise mature microscopically (Fig. 1B, C, Fig. S1D, E). As expected [35], neutrophil elastase was transiently upregulated in cells expressing wt C/EBPα but much less so in C/EBPα-K313 and in C/EBPα-BRM2 cells (Fig. 1D). This phenotype is reminiscent of BRM2 knock-in mice, which showed an age-dependent accumulation of partially differentiated myeloid cells in the bone marrow, but not the periphery [36].

Small differences in the proliferation of progenitor cells were noted (Fig. S2A). During differentiation, cells expressing C/EBPα-K313 but not C/EBPα-BRM2 showed enhanced proliferation, probably due to loss of differentiation (Fig. S2A). Expression of C/EBPα-wt but not C/EBPα-K313 slightly reduced colony size while no differences in colony number were seen (Fig. S2B). While little difference in viability was observed, during differentiation (Fig. S2C), expression of some Bcl-2-family proteins changed as has been reported [23]: anti-apoptotic Bcl-2 and Mcl-1 are downregulated while pro-apoptotic Bim is upregulated. These changes were smaller in C/EBPα-K313 cells; Bim upregulation was reduced in C/EBPα-BRM2 cells again indicating a mild differentiation defect (Fig. S2D).

A lack of GR-1 expression but upregulation of CD11b may also indicate the K313 cells are rather differentiating down the monocytic lineage and not that they have a blockage of differentiation per se, although the lack of c-kit downregulation as well as the reduced regulation of Bcl-2-family proteins argues against this to some extent. In order to analyse this further, progenitor cells and day 4 differentiated cells were analysed for monocyte markers CD14 and CD115. While empty vector controls showed some CD14 expression after differentiation there was no CD115 upregulation (Fig. S3). C/EBPα-wt expression drove higher CD14 expression already at the progenitor stage which was further increased after differentiation. K313 cells showed similar behaviour, but with even more enhanced CD14 expression (Fig. S3A). CD115 was upregulated on day 4 to a similar extent in both empty vector control cells and C/EBPα-wt expressing cells whereas a higher percentage of C/EBPα-k313 cells upregulated it and to a higher level (Fig. S3A). This highlights that there is already some differentiation towards the monocytic lineage in empty vector cells but that this is enhanced by expression of both wild-type C/EBPα and more so by K313 C/EBPα. This suggests that indeed an expression of K313, as well as wild-type C/EBPα, may push a proportion of the progenitors towards a monocytic direction rather than granulocytic, but not the whole population. The cKIT expression and only partial upregulation of CD115 suggest that although there may be some skewing towards monocytic differentiation, the majority of cells expressing either wild type or K313 C/EBPα are not mature monocytes. To test if these cells respond similarly to activation, we treated them with LPS, both at the progenitor and differentiated stages. When stimulated with LPS after 4 days of differentiation C/EBPα-K313 cells produced substantially more TNF and IL-6 than empty vector control or wild-type expressing cells (Fig. S3B). Progenitor cells overexpressing C/EBPα-wt or C/EBPα-K313 secreted TNF while control cells did not. C/EBPα-K313-expressing progenitor cells spontaneously released IL-6 and produced significantly more IL-6 than wt C/EBPα cells after differentiation (Fig. S3B). These results support that there is a more monocyte-like response of the C/EBPα- expressing cells than with the empty vector controls and that this is significantly enhanced by expression of C/EBPα-K313, fitting with the surface marker expression. When comparing the lineage subtypes of different AML-associated mutations, however, there is a clear enrichment of M1 and M2 AML associated with C/EBPα mutations [37], which are rather granulocytic or not yet committed blasts, although it is not clear if any K313 mutations were included in this study.

### C/EBPα-K313 increases cell numbers of Hoxb8-derived cells upon transfer in vivo

**Fig. 2:**
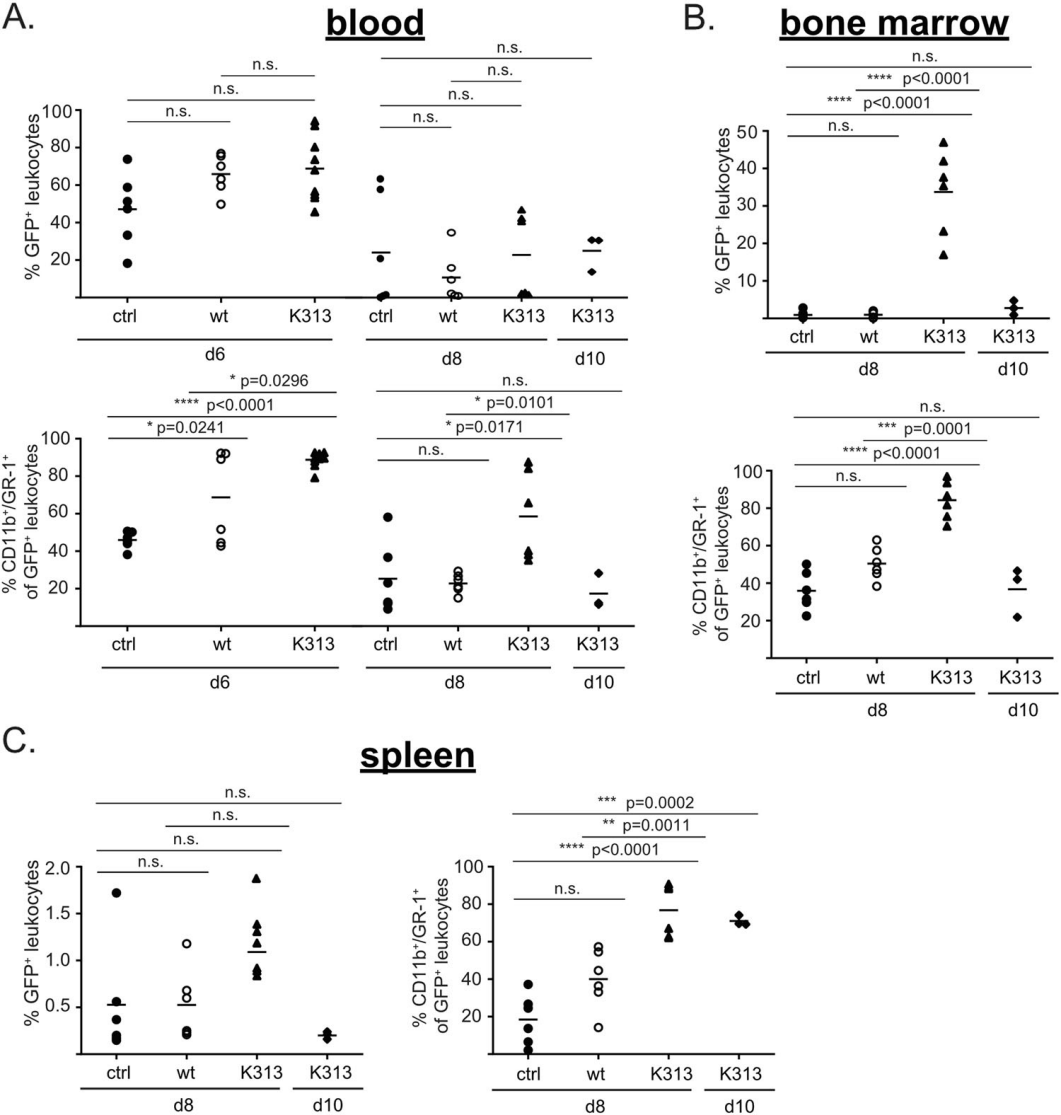
C/EBPα-K313 maintains Hoxb8 neutrophils longer in vivo. The proportion of GFP-positive total leukocytes in blood (**A**), bone marrow (**B**) and spleen (**C**) and percentage of CD11b/Gr-1 double-positive GFP-positive Hoxb8 cells on day 6, 8 and 10 in mice engrafted with empty vector, wt C/EBPα or C/EBPα-K313-expressing Hoxb8 neutrophil progenitors. HoxB8 progenitors were transplanted together with bone marrow cells at a 10:1 ratio. Data represent absolute values and mean of two experiments with three mice per group (n.s.= non-significant, one-way ANOVA with multiple comparisons).

Focussing on C/EBPα-K313 we next adoptively transferred progenitor cells into lethally irradiated mice to determine cell fate in vivo. As described earlier [35], neutrophil progenitors migrated to the bone marrow, and mature neutrophils appeared in peripheral blood and spleen, peaking on d6 after transfer. Mice were analysed on day 6 in peripheral blood and on day 8 in blood, bone marrow and spleen; an additional group that had received C/EBPα-K313 were analysed on day 10. Cell numbers in peripheral blood were similar on day 6 and on day 8 between lines (Fig. 2A). Cells derived from any of the cell lines in peripheral blood mostly expressed CD11b (myeloid cells) but on Day 8 Gr-1 (mature neutrophils) were less present in the empty vector and wild-type expressing cells while the majority were CD11b+ GR-1+ in C/EBPα-K313-expressing cells (Figs. 2A and S4). Stimuli received in vivo appear to allow their full maturation according to those markers. Intriguingly, C/EBPα-K313-expressing CD11b/Gr-1-positive cells persisted longer in bone marrow: substantially more cells were detectable on day 8 (Fig. 2B). Mature C/EBPα-K313-expressing cells were also detected in spleens (Fig. 2C). These cells, therefore, show an altered behaviour in vivo, which might be due to enhanced survival or higher proliferation rates. Given we could not detect the HoxB8 cells longer than the indicated times, we have analysed these time points. It should be noted that at early times after irradiation, there may confounding processes due to the irradiation/reconstitution itself. However, there appears to be a proliferative or survival advantage to the K313-expressing cells at this early stage, even though the results cannot confirm if this may contribute to a leukemic phenotype.

### C/EBPα-K313 lacks DNA-binding activity, but can drive expression of a C/EBP reporter and regulate similar gene networks to endogenous wild-type C/EBPα

The K313 duplication in C/EBPα should block DNA binding, and thus its transcriptional activity. To confirm this, we measured transcriptional activity using a luciferase C/EBP-reporter construct. Reporter activity was low at the progenitor stage and further dropped during differentiation (Fig. S5A). Overexpression of C/EBPα-wt increased reporter activity about 20-fold on day 1 of differentiation (Fig. S5B). Remarkably, C/EBPα-K313 cells showed over 160 fold activity at the same time, and the increased activity was maintained throughout differentiation. This is a surprising finding given the published loss of DNA-binding activity of the K313 mutant. However, it cannot distinguish between direct binding and more indirect effects such as forming complexes with other transcription factors. We, therefore, performed chromatin immunoprecipitation (CHIP) experiments using the FLAG-tagged C/EBPα-expressing cells and anti-FLAG antibodies to test for DNA binding of the mutant. These CHIP assays only identify if the p42 version of C/EBPα is binding DNA as the FLAG tag is N-terminal. qPCR was performed against the immune-precipitated DNA, and three transcriptional targets of C/EBPα (C/EBPα itself, PTGS2 and IL6Ra) were analysed for enrichment. Comparing with empty vector controls, FLAG-C/EBPα showed a consistent enrichment at all three loci, however, there was a complete lack of enrichment for FLAG-K313-C/EBPα (Fig. S5C). This confirms that the K313 mutation disrupts DNA binding, at least for the p42 isoform.

CHIP was also performed using the C/EBP Luciferase reporter cells to determine if C/EBPα enriched for the reporter construct too. Wild-type C/EBPα enriched the luciferase reporter construct, but in contradiction to the CHIP using endogenous targets, the K313 mutant was enriched, even more so than the wild type (Fig. S5D). PTGS2 again showed enrichment in wild-type cells, but not in K313-expressing cells (Fig. S5D). The mutant could therefore bind to the reporter construct directly, or more likely is recruited through another C/EBP family member. There was however no significant upregulation of other C/EBP family members in the K313-expressing cell lines at the mRNA level (data not shown).

Further support for a lack of DNA binding in the majority of C/EBPα targets can be seen using a previously published CHIP-Seq data set from FDCP1 cells expressing either wild type or K313-mutated C/EBPα [31]. Looking at the global peaks detected in the K313-expressing cells compared with wild type, there is a significant reduction in peak intensity and number (Fig. S5E), thus confirming the lack of effective DNA binding of the K313 mutated construct. There are however some peaks detectable for the mutant, both at sites also bound by wild-type C/EBPα as well as sites that appear to be specific to the mutant (Fig. S5E). A closer look at the peaks detected for PTGS2, confirms that the peaks present in wild-type c/EBPα expressing cells are absent in the K313-expressing cells further confirming our CHIP findings (Fig. S5E).

**Fig. 3:**
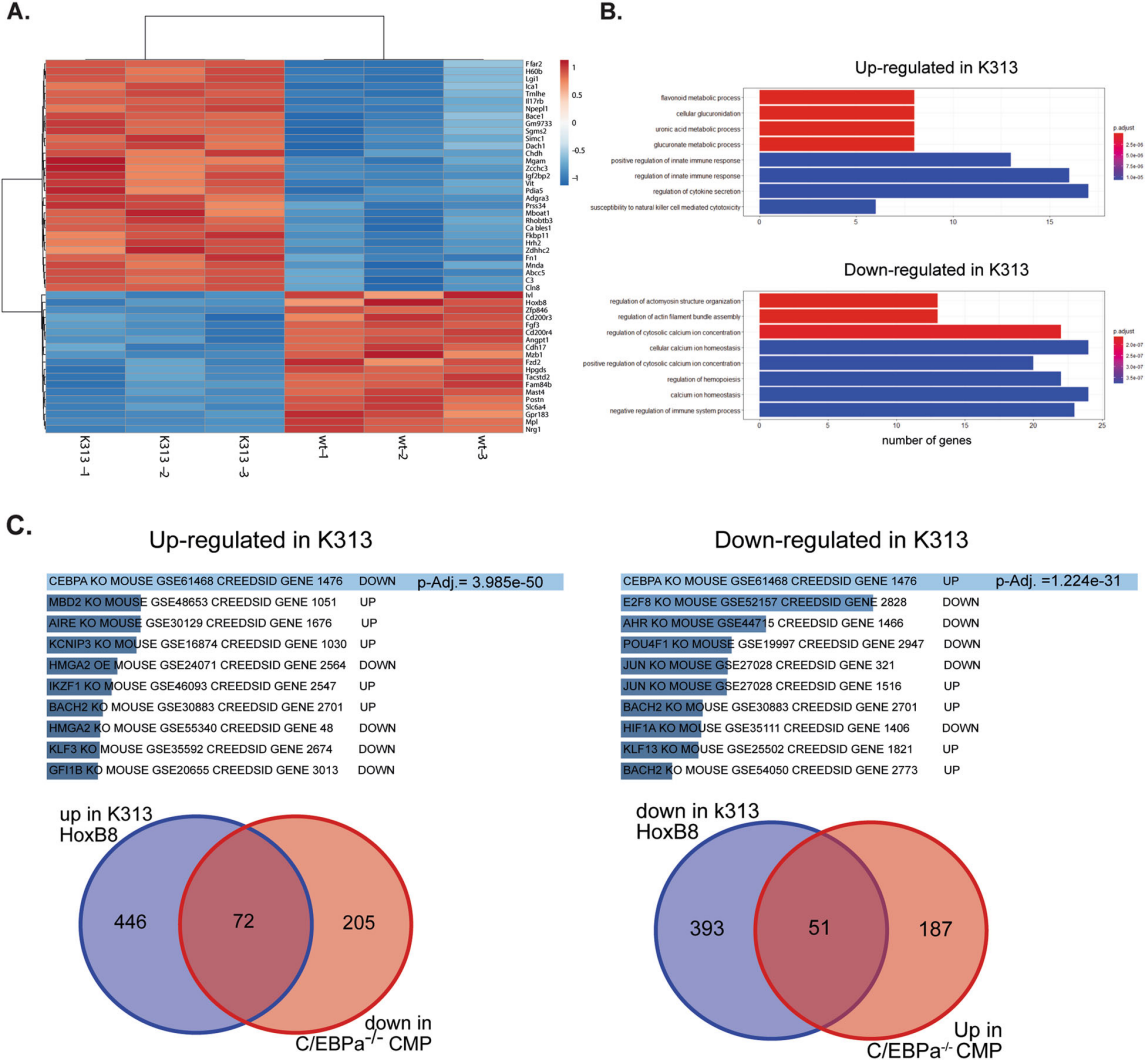
Differentiating HoxB8 neutrophil progenitors show altered gene expression with C/EBPα-K313 vs. C/EBPα-wt expression. **A** HoxB8 progenitors were differentiated for 1 day before RNA was extracted and assayed for gene expression using an affymterix gene chip. Heatmap showing the relative expression of the 50 most significantly differentially expressed genes (DEGs) from three samples of wild type and C/EBPa-K313-expressing cells. **B** Bar chart showing the most significant terms in the GO:BP analyses for upregulated and downregulated genes in K313 mutant C/EBPa expressing cells. The *x* axis refers to the number of enriched genes in the corresponding function. **C** Results from ENRICHR suite analysis for transcription factor perturbations for genes upregulated or downregulated in K313 C/EBPα-expressing cells. Indicated are the top hits for transcription factor knockouts and if the genes were up- or downregulated in the respective knockouts. Also shown are Venn diagrams showing the overlap of genes in the indicated lists.

To determine what difference this activity of K313-C/EBPα has on gene expression, microarray analysis of RNA isolated from cells expressing wt or K313-C/EBPα (day 1 of differentiation) was performed, and the expression in C/EBPα-K313 vs wild-type was compared. The top 50 differentially expressed genes and principal component analysis showed that wt and C/EBPα-K313 cells had distinct transcriptional profiles ((Fig. 3A and Fig. S6A). Microarray results were validated using qPCR with specific primers for two down and two upregulated genes and confirmed the results obtained with the microarray (Fig. S6B). The expression of two monocytic markers CD14 and S100A9 was increased confirming the surface expression of CD14 and further supporting that expression of C/EBPα-K313 is shifting differentiation towards a monocytic direction. The up and downregulated gene lists were analysed for GO pathway enrichment (Fig. 3B). Genes involved in hemopoiesis were downregulated in C/EBPα-K313-expressing cells along with genes regulating calcium homoeostasis and negative regulation of immune system processes (Fig. 3C). Upregulated genes were largely associated with various metabolic pathways as well as increased immune system processes and cytokine secretion. This aligns with a block in differentiation, at least in some of the cells, and potentially also a skewing towards a more monocytic differentiation.

To explain the discrepancies seen with reporter activity and CHIP results, we further analysed the microarray gene sets for transcription factor associations for other factors that may be regulating gene expression. When the upregulated genes were analysed using the ENRICHR suite to determine similar changes in cells with transcription factor-deficiencies [38, 39], the best hit for gene enrichment after transcription factor perturbations was for genes downregulated in C/EBPα-deficient cells (Fig. 3C). Conversely, genes that were downregulated in the Hoxb8 C/EBPα-K313-expressing cells matched most closely with genes upregulated in C/EBPα deficient cells. Comparison of the gene sets from the knockout cell study and our overexpression study showed substantial overlap in the regulated genes (Fig. 3D). Thus, expression of C/EBPα-K313 drives a transcriptional response inverse to the deletion of C/EBPα indicating that it regulates a similar transcriptional profile to the wild-type endogenous protein, despite its apparent lack of DNA-binding activity.

To further understand which transcription factors are associated with C/EBPα-K313 activity, we analysed genes downregulated in C/EBPα deficient CMPs generated in a previous study [30] (GSE61468) for transcription factor binding using the CHEA3 tool [29]. C/EBPα did not appear as a common factor, but instead, the top hit was SPI1/PU.1 (Fig. S7). When the list of upregulated genes in C/EBPα-K313-expressing cells was analysed, similar to the genes downregulated in C/EBPα KO CMPs, SPI1/PU.1 is high on the list. Network analysis with the CHEA3 tool identified SPI1/PU.1 as a highly connected node within both networks, suggesting it plays an important regulatory role within both gene sets (Fig. S7). Interestingly, another highly connected node in the network is IKZF1. IKZF1 is also mutated in AML and associated with C/EBPα mutations, but the significance of this association is unclear [40, 41].

Analysis of transcription factor target genes using the upregulated genes in C/EBPα-deficient CMP’s further revealed a strong link to the FOS/JUN transcription factor family (Fig. S7). AP1 transcription factors can hetero-dimerise with C/EBPα, resulting in the recognition of hybrid recognition motifs [42]. In addition, C/EBPα downregulates cJUN in order to regulate granulocytic differentiation [43], thus supporting the observed increase in AP1 target genes in the study of C/EBPα-deficient CMPs and confirming the analysis is able to identify enriched transcription factors. Transcription factor enrichment in genes downregulated in C/EBPα-K313-expressing cells also identified SPI1/PU.1, again within the top four factors and constituted a central node within the local network. The data support the conclusion that, despite the loss of DNA-binding activity, C/EBPα-K313 is actively regulating a similar set of genes to wild-type C/EBPα likely also through co-regulating other factors such as SPI1/PU.1. The expression of PU.1 was not strongly affected by expression of wild-type or K313 C/EBPα (Fig. S8), suggesting that regulation of its activity, rather than expression levels is the mechanism behind this effect.

### The K313-mutation increases C/EBPα protein levels

**Fig. 4:**
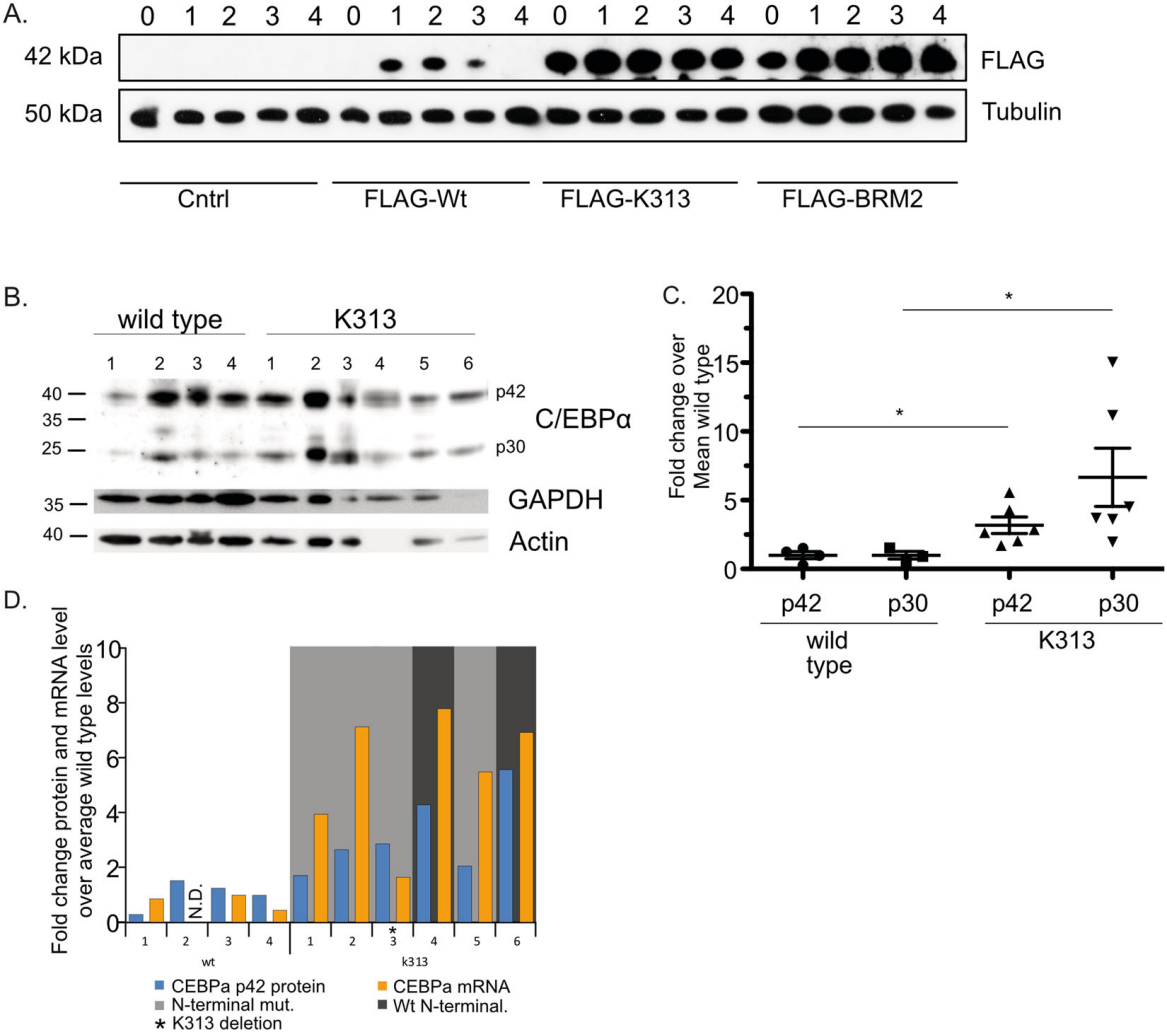
C/EBPα-K313 and C/EBPα-BRM2 are expressed at higher levels than wild-type C/EBPα. **A** HoxB8 neutrophil progenitor cells expressing the indicated C/EBPα constructs were differentiated and samples were taken for western blot. Levels of C/EBPα were shown. **B** Primary human AML samples without (wild type) or with K313 mutations were analysed by western blot for C/EBPα levels. Both GAPDH and β-actin were used as loading controls. See table S1 for the specific mutations. **C** Levels of C/EBPα isoforms were quantified for the indicated samples from **B**. and are shown as fold change over wild-type levels after normalising to loading controls. Mean is shown with error bars indicating standard error of mean. **D** Comparison of C/EBPα mRNA levels to protein levels in human AML samples from **B**. Indicated are whether or not N-terminal C/EBPα mutations are also present.

While analysing the expression of the C/EBPα wild type and mutants, we noticed a striking difference in expression levels of wt and mutant C/EBPα proteins. Expression of C/EBPα-K313 protein was massively increased, as was C/EBPα-BRM2 hinting that this may be a feature of some C-terminal mutations (Fig. 4A). This increase in C/EBPα-K313 was independent of mRNA levels as both GFP-fluorescence (GFP is expressed from an IRES) was similar between the cells (Fig. S9A) and quantification of C/EBPα-mRNA by qPCR showed no increase of C/EBPα-K313 mRNA over wild type (Fig. S9B). To test if this is due to the increased half-life of C/EBPα, we blocked protein synthesis with cycloheximide and monitored C/EBPα-protein levels. Bcl-X_L_ was co-expressed to prevent cycloheximide-induced apoptosis. We found very little difference in protein stability (Fig. S9C, D) showing that the higher protein levels are not due to increased stability.

To validate our findings and to test if a similar activity is present in human AML patients with C/EBPα mutations, we compared human AML-blast cells containing the C/EBPα-K313 mutation with AML-blast cells with wt C/EBPα (Table S1). AML C/EBPα-K313 samples from six patients were compared with four patient samples with wt C/EBPα (Fig. 4B). The K313 mutants showed elevated C/EBPα-protein levels compared to wild-type expressing cells (Fig. 4B, C). Remarkably, both the p42 and p30 isoforms were upregulated, regardless of the presence or absence of additional N-terminal mutations on the second allele (Fig. 4C, Table S1). There was a clear increase in C/EBPα mRNA in the K313-mutated blasts (Fig. 4D). The level of protein expression was not uniformly dependent on mRNA expression, however, suggesting that other mechanisms may also be at play. One of the C/EBPα-mutations in the AML blasts (sample #3) was a deletion of K313, not a duplication, and cells expressing this mutant showed only slightly elevated mRNA but a nearly threefold increase in p42 and an 11-fold increase in p30 C/EBPα (Fig. 4B–D). While the contribution of each allele to the mRNA levels cannot be differentiated, in those N-terminal mutant bearing C/EBPα blasts (samples 1–3 and 5), all of the p42 protein isoforms must come from the K313 mutant allele. In the two monoallelic K313 samples additional p42 C/EBPα expression from the wild-type allele likely explains the higher p42 C/EBPα protein levels above the N-terminal harbouring mutants. C/EBPα is known to bind to its own promoter, suggesting it likely regulates its own expression. This may explain the increased mRNA levels in the C/EBPα mutant AML blasts [44]. The results strongly suggest that increased expression of C/EBPα-K313 is also a feature of human C/EBPα-K313 mutant blasts. Together, these data show there is an upregulation of C/EBPα-K313 expression that may include regulation at both the translational and transcriptional levels and the enhanced and prolonged-expression of C/EBPα target gene expression in progenitor cells likely contributes to the leukaemia promoting effects of K313 mutations.

## Discussion

In vivo transplantation of C/EBPα-K313-expressing Hoxb8 cells could not induce leukaemia. Instead, cells appeared to proliferate more, albeit in an artificial setting, and were maintained for several days longer than wild-type expressing cells, and however, did differentiate into mature neutrophils. Mouse knock-in models expressing C/EBPα-k313 [45] also showed only mild phenotype relative to p30 knock-in mice [45, 46] and it must be considered that these models express the mutant proteins from the earliest possible stage while this HoxB8 model introduces C/EBPα into cells that are likely at a more committed stage of differentiation. Despite this lack of oncogenic ability, the HoxB8 model was able to reveal new levels of regulation through mutation of K313 in C/EBPα.

K313-duplication mutants showed higher activity in driving reporter expression as well as a C/EBPα transcriptional programme. We could confirm a failure of C/EBPα-K313 to bind DNA [47] at endogenous loci, but show significant binding to a consensus reporter construct, probably through interaction with other C/EBP family members. Binding at other loci could also be identified using CHIP-seq confirming that the mutant can be recruited to DNA. Transcription factor enrichment analysis suggests that the transcriptional programme activated by C/EBPα (both wild type and K313 mutants), is heavily enriched for SPI1/PU.1 target genes. C/EBPα is known to regulate the activity of SPI1/PU.1 as well as the AP1 family of transcription factors to regulate monopoiesis [48]. C/EBPΑ can regulate enhancers important in GMP differentiation in coordination with SPI1/PU.1. Specifically, C/EBPα has a pioneering function at enhancers involved in differentiation of GMPs and promotes recruitment of SPI1/PU.1 as well as regulating SPI1/PU.1 levels [49]. C/EBPα overexpression in HoxB8 progenitor cells did not increase the expression of PU.1, suggesting that the increased activity of PU.1 is through another mechanism. Loss of pioneering functions of CEBPα-K313 may be the cause of downregulation of some PU.1 target genes detected in the transcription factor enrichment analysis. C/EBPα proteins were also previously shown to cooperate with NF-κB p50, thereby inducing the expression of several target genes [50–54]. The complex interplay of C/EBPα with other transcription factors likely does not require DNA binding for all levels of regulation, and we propose that despite the loss of direct DNA binding of K313 mutant C/EBPα, it is still functional in driving expression of a large number of genes involved in myeloid differentiation through these regulatory interactions with other factors, particularly PU.1.

It was previously shown using knock-in mice that sites that are common to both p30 and p42 isoforms, as well as those that specifically recruit p30 overlap strongly with ETS family transcription factors including SPI1/PU.1, whereas those sites preferentially recruiting the p42 isoform, are depleted for these factors [55, 56]. A striking feature of the K313 mutations was the aberrantly high protein expression, including the p30 form, which correlated with reporter activity and regulation of C/EBPα target gene expression. Together, this may suggest that it is rather the p30 form of C/EBPα-K313 that is active in driving SPI1/PU.1-dependent gene expression, even when it contains the K313 mutation. It should be pointed out that the HoxB8 cells express endogenous C/EBPα too and we cannot rule out that expression of the K313 mutant somehow leads to increased activity of this fraction of C/EBPα.

Expression of C/EBPα, either wild type or particularly C/EBPα-K313 led to the upregulation of monocytic markers such as CD14 and CD115. Expression of ER-fusions of C/EBPα promotes monopoiesis [57]. It is likely that similar activity is seen here for C/EBPα-K313, however, based on staining of cKIT and CXCR2 these cells are not mature monocytes. Prolonged activity of C/EBPα likely skews the progenitors towards monocytic commitment, but also keeps them in an immature form, whereas a normal reduction in C/EBPα expression promotes differentiation. A number of other differentiation defects in K313 cells were apparent (neutrophil elastase, Bcl-2-family members, surface markers; morphological changes; continued proliferation). Some genes in particular that showed altered expression in our analysis are candidates for inhibitors of differentiation, such as Id2 [58], Zfp36l1 [59] or Gfi1b [60], which were all downregulated. Pro-inflammatory activity was higher in K313 cells probably due to the monocytic phenotype and upregulation of CD14. This propensity for exaggerated inflammatory responses including spontaneous IL-6 secretion may also contribute to disease if it also replicated in human AML.

What is leading to the strong overexpression of K313 mutant C/EBPα is not clear. C/EBPα translation can be regulated at multiple levels and perturbations in these regulatory mechanisms are also associated with AML (reviewed in [61]). Indeed, higher levels of expression were also seen with human K313-expressing blasts. Particularly interesting was the upregulation p30 C/EBPα. N-terminal mutations in C/EBPα leading to expression of only the p30 isoform are common and our study suggests that mutation of K313 in the C-terminal region (deletion or duplication) also leads to significant upregulation of the p30 isoform. This may be a significant activity of the K313 mutation and effectively increase the relative dosage of p30 to p42, although the activity of p30 C/EBPα with C-terminal mutations is not clear and to date not well studied. In HoxB8 cells, although p30 was upregulated, it was not to the same extent as in human AML blasts. This may be owing to the construct used lacking aspects of translational control such as a well characterised µORF directly upstream of the C/EBPα ORF [62]. Regardless of the mechanism of upregulation, this study suggests that mutation in residue K313 (duplications or deletions) lead to strongly enhanced production of C/EBPα including the p30 isoform, which likely contributes to its oncogenic activity.

The Hoxb8-model represents a valuable tool to study the pathophysiology of human AML. In the case of C/EBPα mutations, the model reproduces impaired differentiation and cellular expansion in various organs, both of which are key features of human AML despite falling short of inducing AML on its own. With its ease of cell generation, genetic manipulation and cell biological study, this model appears to be a valuable addition to the toolbox for the study of malignant granulopoiesis. The model also permits the generation of other cell lineages such as macrophages [22] and lymphoid-primed multipotent progenitor cells [63], and may thus prove useful for the study of other haematopoietic malignancies.

## Supplementary information

Supplemental figures
