# Supplementary material for: The AML-associated K313 mutation enhances C/EBPα activity by leading to C/EBPα overexpression
Source: Cell Death Dis. 2021 Jul 5;12(7):675. doi: 10.1038/s41419-021-03948-6 (PMC8257693; doi:10.1038/s41419-021-03948-6)
Supplement: Supplementary file 1 — Supplemental figures [file 41419_2021_3948_MOESM1_ESM.pdf]

## Supplemental Table 1: Human AML samples and associated mutations.

| Group | Sample | N-terminal CF7/CR7 500-844 | N-terminal CF14/CR13 744-1045 | Middle CF9/CR9 998-1441 | C-terminal CF10/CR10 1312-1726 | Age (Years) | Sex | BM Blasts (%) | PB Blasts (%) | FLT3-ITD | FLT3-TKD | NPM1 | PCR_8_21 | PCR_INV1 | PCR_15_1 | PCR_9_11 |
|---|---|---|---|---|---|---|---|---|---|---|---|---|---|---|---|---|
| C/EBPα mutants | 1 | 704_705insGCGCGGG,G38fsx108,N-terminal stop | - | 1158C>A,silent mutation | 1527_1528insCAG,Q312_K313insQ, insertion in ZIP | 55 | F | | | - | - | - | - | - | - | - |
| | 2 | 797_798insTA,D69fsx159, N-terminal stop | - | 1175_1180dup | 1530_1531insAAG,K313Q314insK(K313dup), insertion in ZIP | 48 | F | 95.5 | 97 | + | + | - | - | - | - | - |
| | 3 | 695_707del,R35fsX154,N-terminal stop | - | - | 1528_1530delAAG, K313del,deletion in ZIP | 47 | F | | | - | - | - | - | - | - | - |
| | 4 | - | - | - | 1528_1530dup, K313dup,duplication in ZIP | 36 | F | 90 | | - | - | - | - | - | - | - |
| | 5 | - | 800delC,D69fsx158,N-terminal stop | 1175_1180dup | 1528_1530dupAAG, K313dup,duplication in ZIP | 34 | M | 45 | 38 | - | - | - | - | - | - | - |
| | 6 | - | - | - | 1528_1530dup,K313dup, duplication in ZIP | 63 | F | | | - | - | - | - | - | - | - |
| C/EBPα wild type | 1 | - | - | - | - | 56 | M | 58.5 | 9 | - | - | - | - | + | - | - |
| | 2 | - | - | - | - | 41 | F | 65 | 38 | - | - | - | - | - | - | + |
| | 3 | - | - | - | - | 44 | F | | | - | - | + | - | - | - | - |
| | 4 | - | - | - | - | 40 | F | | | - | - | - | - | - | - | - |

## Supplementary figure legends

**Figure S1: Gr-1, CD-11b and c-kit surface expression of HoxB8 neutrophil progenitors during differentiation.**

**A.** Wild type HoxB8 neutrophil progenitors were cultured and differentiated as described in methods for the indicated number of days and surface expression of Cd-11b, GR1 and c-kit were analysed by flow cytometry. **B.** Schematic of constructs expressed in wt HoxB8 neutrophil progenitors. **C.** Surface staining with antibodies against Gr-1 and CD11b. HoxB8 progenitors expressing wild type, p42 or p30 isoforms were differentiated for 4 days, stained for Gr-1 and CD11b. **D.** Loss of Gr-1 and CD11b during differentiation of progenitor cells expressing C/EBPα wt, C/EBPα-K313, C/EBPα-BRM2. Progenitor cells were differentiated for 4 days, stained for Gr-1 and CD11b. Quantifications show mean and SEM of at least three independent experiments. P values were calculated using one way ANOVA with multiple comparisons. **E.** Loss of Gr-1 and CD11b during differentiation of wt progenitor cells expressing FLAG tagged C/EBPα–wt, C/EBPα-K313, C/EBPα-BRM2. Progenitor cells were differentiated for 4 days, stained for Gr-1 and CD11b.

**Figure S2: Proliferation of HoxB8 progenitors is altered by expression of C/EBPα-K313.**

**A.** C/EBPα-K313 expression promotes proliferation during differentiation, but not at progenitor stage. Proliferation of Hoxb8 neutrophil progenitor (left panel) or differentiating (right panel) cells expressing empty vector or FLAG-C/EBPα-wt, K313 or BRM2 was assessed by counting cells each day. Asterisks show significance between C/EBPα-K313 and C/EBPα-wt expressing cells (* p<0.05, ** p<0.01, *** p<0.001, One way ANOVA with multiple comparisons) **B**. **(left panel)** HoxB8 progenitors expressing the indicated constructs were cultured in methylcellulose media containing -estradiol to prevent differentiation for 7 days. Box plots show

median of colony size (line inside box), upper and lower quartile of colonies (box below and above the median line) and minimal and maximal size of colonies (whiskers). Means are indicated by a plus symbol (+). Data are from three independent experiments. **(Right panel)** Colony numbers were also counted after 7 days. Data represent mean and SEM of three independent experiments (n.s.= non-significant, one-way ANOVA). **C.** Survival during differentiation. HoxB8 progenitor cells expressing the indicated C/EBPα constructs were induced to differentiate and survival was assessed by propidium iodide exclusion. Asterisks show significance between C/EBPα-K313 and C/EBPα-wt expressing cells (* p<0.05, ** p<0.01, *** p<0.001, One-way ANOVA with multiple comparisons) **D.** Expression of BCL-2 family proteins during differentiation. HoxB8 progenitor cells expressing the indicated C/EBPα constructs were induced to differentiate and cell extracts were analysed by Western blot. All error bars represent SEM from at least three independent experiments.

**Figure S3: C/EBP -K313** overexpressing cells show upregulation of monocytic markers

**(A**) HoxB8 progenitor cells or day 4 differentiated cells expressing either empty vector, wild type C/EBPα or C/EBPα-K313 were stained for CD14, CD115 and CXCR2 and analysed by flow cytometry. Shown are representative histograms of surface expression of the indicated markers. Graphs show the mean Mean Fluoresence Intensity (MFI) from at least 4 independent experiments. Error bars are SEM and significance was calculated using One way ANOVA with multiple comparisons (Tukey).

**(B)** ELISA showing levels of TNF and IL-6 in Hoxb8 progenitors and day 4 differentiated neutrophils expressing empty vector, wt, or C/EBPα-K313. Cells were stimulated with 1µg/ml LPS for 8 h followed by ELISA to measure secreted cytokine levels. Data show mean and SEM of at least three independent experiments.

**Figure S4: Representative FACS polots of HoxB8 cells adoptively transferered into irradiated mice.**

Example plots are shown for single mice analysed on Day 8 for HoxB8 (GFP+) cells adoptively transferred into lethally irradiated mice as described in the methods section. GFP+ cells were analysed for GR-1 and CD11b expression.

**Figure S5:** C/EBP -K313 can activate a C/EBP reporter, but the p42 isoform lacks DNA binding activity.

**A-B.** Activation of the C/EBP responsive element was measured by firefly luciferase activity in live cells after incubation with D-luciferin in cells expressing reporter only (A.) and also in wt Hoxb8 neutrophils expressing the C/EBP reporter as well as the indicated C/EBPα constructs during differentiation (d0-d4) (B.). Activity in the overexpressing cells is shown as fold-induction relative to reporter only cells. Data show mean and SEM of five independent experiments with duplicate samples each. **C.** Chromatin Immuno Precipitation (CHIP) was performed as described in the methods section using anti FLAG antibodies to pull down tagged C/EBPα. Enrichment was normalized to the empty vector control cells and then internally to the SFI3 locus Shown are the means and error bars are SEM of three independent experiments. P values were calculated using t-test. **D.** FLAG-CEBPa CHIP against C/CEBP reporter. CHIP was performed as in (C) but using cells expressing the C/EBP-luciferase reporter and qPCR was performed to quantify enrichment of the reporter DNA as well as PTGS2 as a positive control. Enrichment was normalized to the empty vector control cells and then internally to the SFI3 locus. Shown are the means and error bars are SEM of at least three independent experiments. P values were calculated using t-test. **E.** Top panel - Track of the wild type C/EBPα superimposed peak signals on both strands (intensity on Y-axis) at 6917 peaks. the X-axis represents the 20000 bp surrounding the each region and was segmented in to 400 bins and smoothed for 1 bins. A bar showing

the relationship between colouring and the overlayed signal can be seen right side of the plot. Note that regions that are close to neighbouring regions (within a distance corresponding to the visualized window) will be depicted more than once. Bottom Panel – as above but for the 219 C/EBP$\alpha$-K313 peaks detected. Right panel – Peak tracks or each sample at the PTGS2 gene locus. The peak detected by the FLAG-CHIP is indicated (red bar).

**Figure S6: Microarray validation**

PCA analysis of the three wild type and three C/EBP -K313 expressing samples, show that they cluster into separate groups. **B.** qPCR was performed to quantify the indicated genes, two from down regulated genes in the microarray and two from upregulated genes. Shown are means from three independent experiments error bars are SEM. The corresponding fold change detected in the microarray is also shown as a comparison.

**Figure S7: Transcription factor enrichment analysis of differentially regulated genes in HoxB8 C/EBP overexpressing cells and comparison to C/EBP knockout CMP differentially regulated genes.**

Gene lists of up or down regulated in C/EBP -K313 expressing HoxB8 cells and gene lists of up or down regulated genes in C/EBP Knockout CMP mice from a previously published study were analysed using the CHEA3 tool. Shown are the top 10 enriched transcription factors for each list ranked using the Mean based Rank score from the tool. Local network analysis within the CHEA3 tool is also shown.

**Figure S8:PU.1 is not upregulated by c/EBP$\alpha$ overexpression.**

HoxB8 progenitors were lysed in 1x SDS sample buffer and separated by SDS-PAGE followed by western blotting for PU.1 and c/EBP$\alpha$ with actin as a loading control.

**Figure S9: C/EBP K313 is not upregulated at the transcriptional level or by protein stability in HoxB8 cells.**

**A.** Expression of GFP in HoxB8 cells expressing the indicated C/EBP constructs was measured by flow cytometry in progenitors and on day 4 of differentiation. **B.** Relative mRNA levels of C/EBP in cells expressing the indicated C/EBP constructs were analysed by qRT-PCR (normalised to β-Actin) on day 1 and day 2 of differentiation. Control refers to wt Hoxb8 neutrophils. Data represent mean and SEM of three independent experiments. **C.** HoxB8 cells expressing wild type C/EBP or C/EBP - K313 as well as Bcl-XL were differentiated for 1 day and treated with cycloheximide (10 g/ml) for the indicated times. Cell lysates were taken and analysed for C/EBP expression by western blot. **D.** Densitometry of the western blots shown in (A) was performed. Data are from one experiment that is representative of at least three independent.

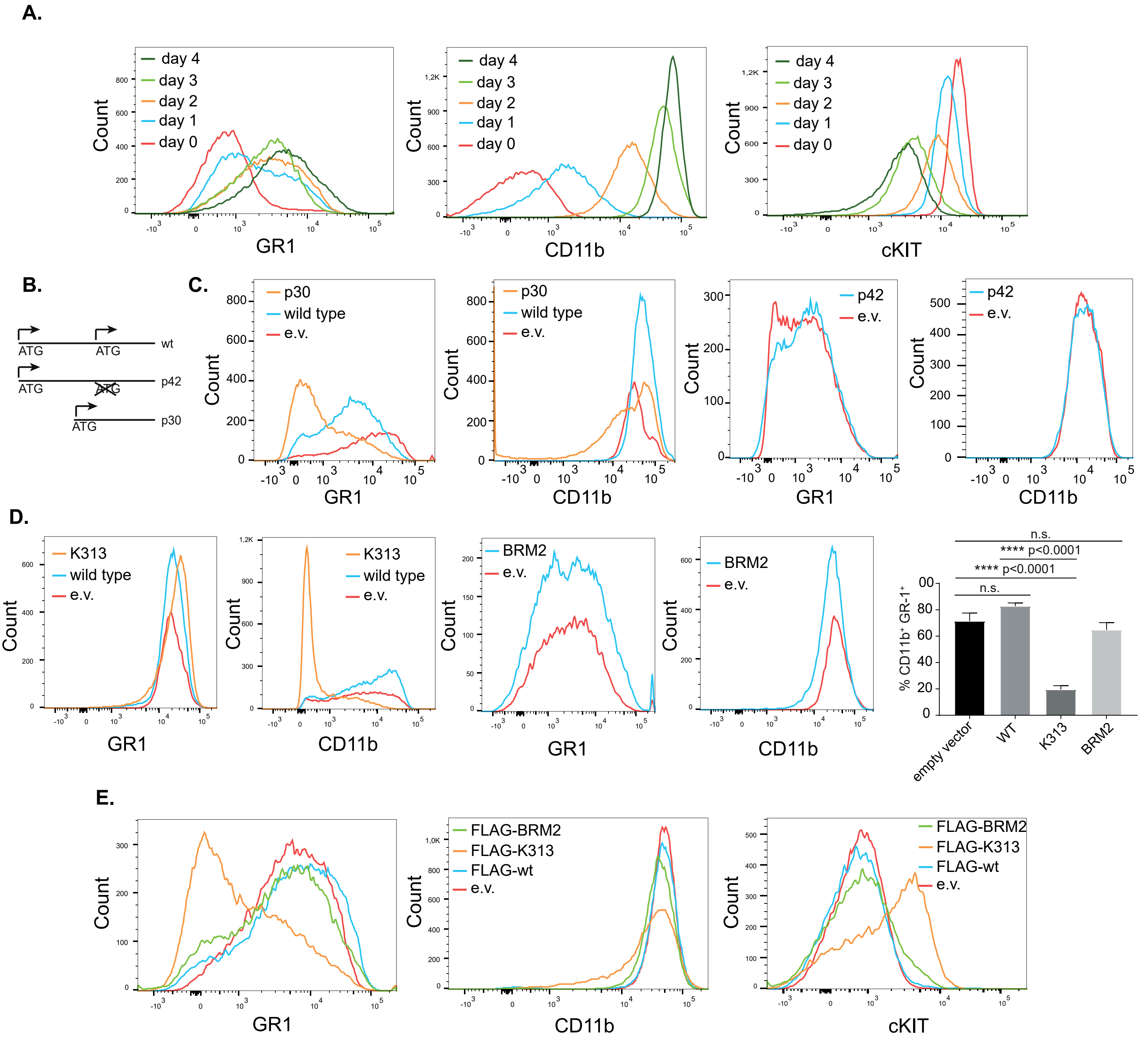
A.
day 4
day 3
day 2
day 1
day 0
Count
GR1
CD11b
cKIT
B.
ATG
ATG
wt
ATG
ATG
p42
ATG
p30
C.
p30
wild type
e.v.
p42
e.v.
Count
GR1
CD11b
D.
K313
wild type
e.v.
BRM2
e.v.
Count
GR1
CD11b
n.s.
**** p<0.0001
**** p<0.0001
n.s.
% CD11b⁺ GR-1⁺
empty vector
WT
K313
BRM2
E.
FLAG-BRM2
FLAG-K313
FLAG-wt
e.v.
Count
GR1
CD11b
cKIT

Fig. S1

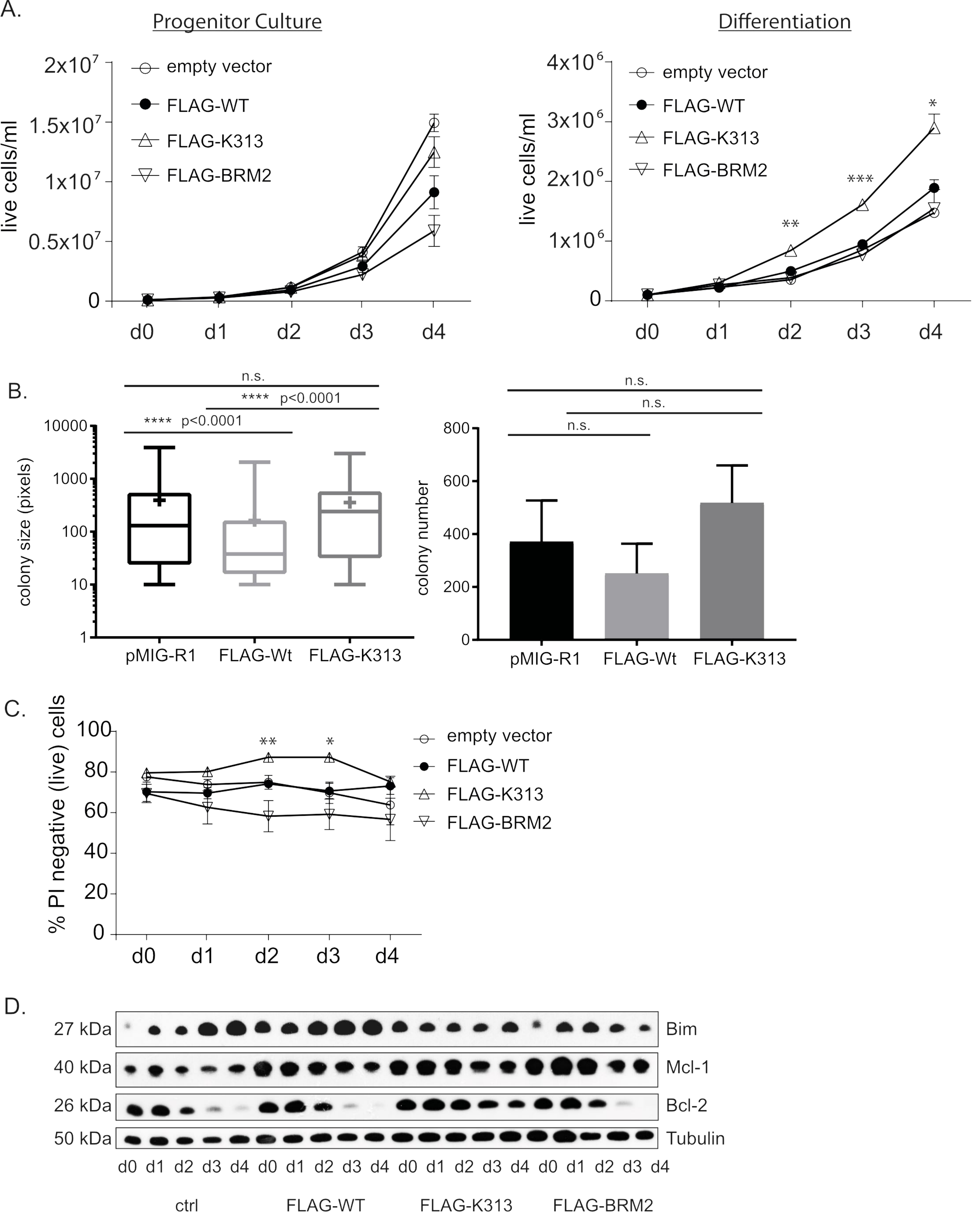

A.
Progenitor Culture
Differentiation
live cells/ml
2x10^7
1.5x10^7
1x10^7
0.5x10^7
0
4x10^6
3x10^6
2x10^6
1x10^6
empty vector
FLAG-WT
FLAG-K313
FLAG-BRM2
**
***
*
d0
d1
d2
d3
d4
B.
n.s.
**** p<0.0001
**** p<0.0001
colony size (pixels)
10000
1000
100
10
1
pMIG-R1
FLAG-Wt
FLAG-K313
colony number
800
600
400
200
C.
% PI negative (live) cells
100
80
60
40
20
D.
27 kDa
40 kDa
26 kDa
50 kDa
Bim
Mcl-1
Bcl-2
Tubulin
ctrl


Fig. S2

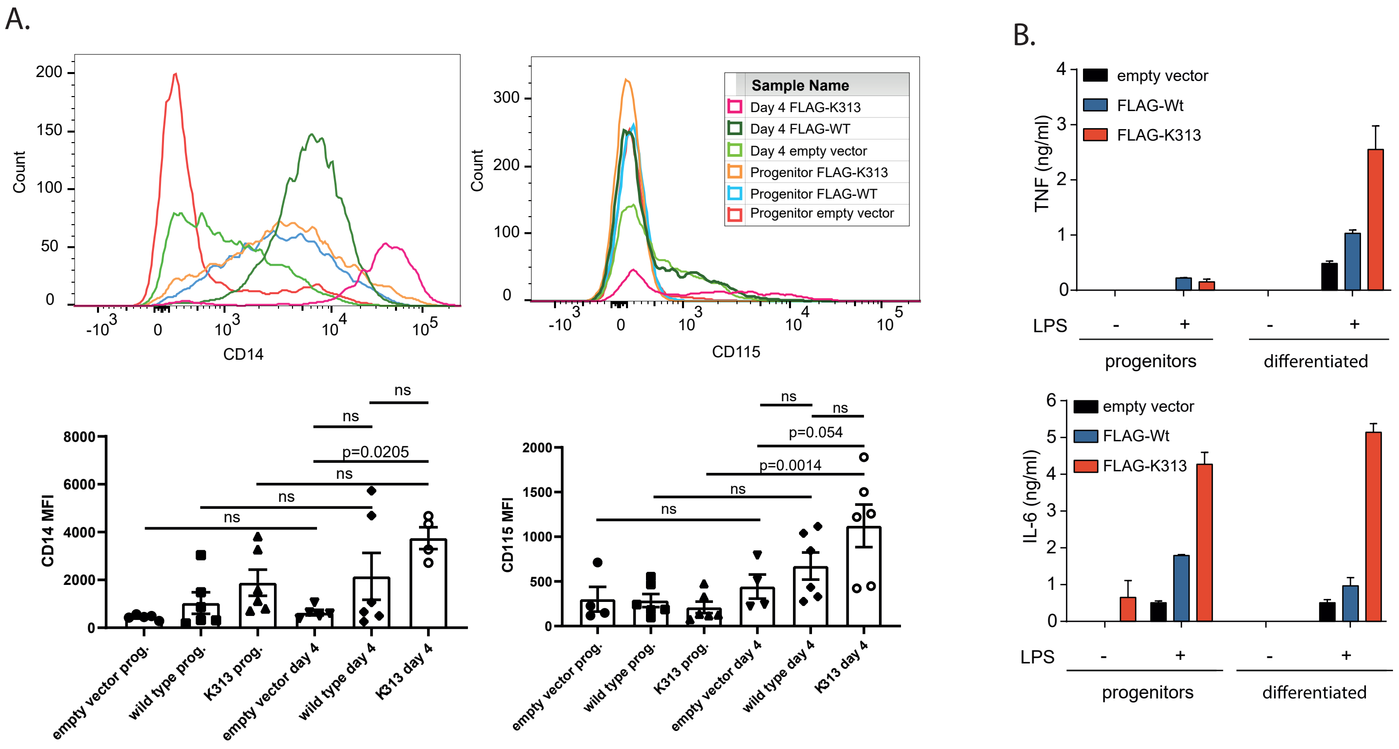
A.
Count
CD14
Count
CD115
Sample Name
Day 4 FLAG-K313
Day 4 FLAG-WT
Day 4 empty vector
Progenitor FLAG-K313
Progenitor FLAG-WT
Progenitor empty vector
CD14 MFI
ns
p=0.0205
empty vector prog.
wild type prog.
K313 prog.
empty vector day 4
wild type day 4
K313 day 4
CD115 MFI
p=0.054
p=0.0014
B.
TNF (ng/ml)
empty vector
FLAG-Wt
FLAG-K313
LPS
progenitors
differentiated
IL-6 (ng/ml)

Figure S3

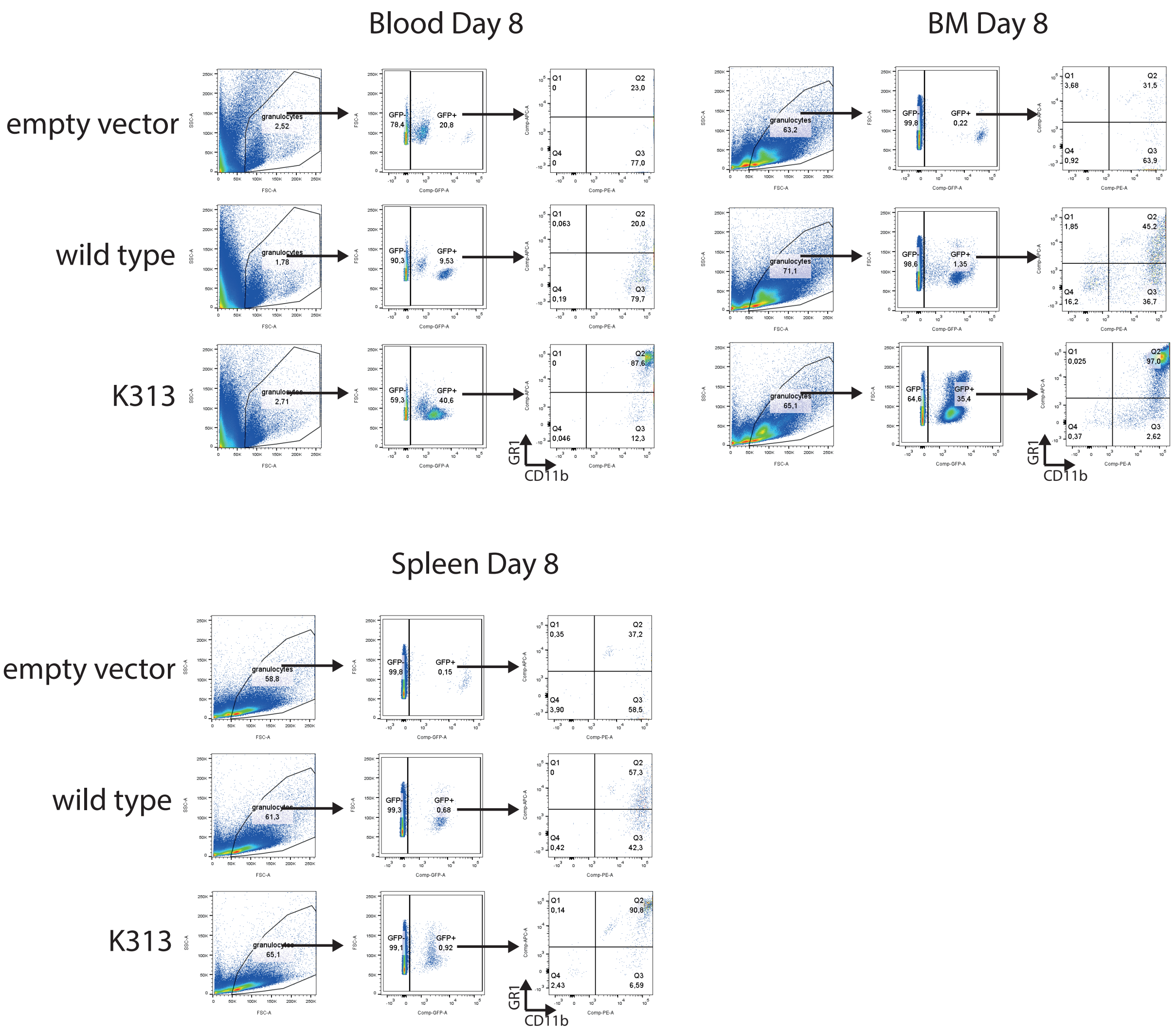
Blood Day 8
BM Day 8
empty vector
wild type
K313
GR1
CD11b
Spleen Day 8
empty vector
wild type
K313
GR1
CD11b

Fig. S4

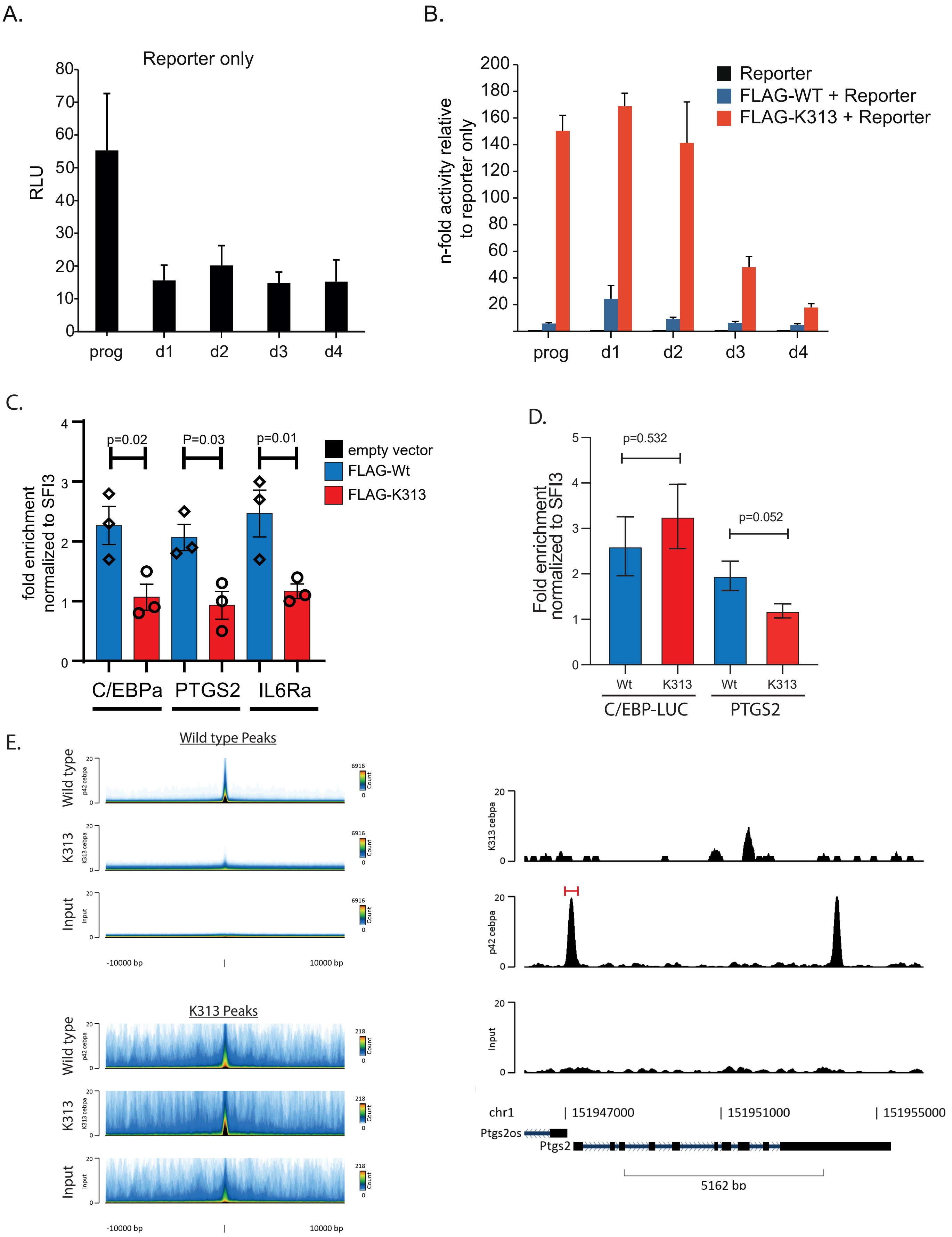
A.
Reporter only
RLU
prog
d1
d2
d3
d4
B.
n-fold activity relative to reporter only
Reporter
FLAG-WT + Reporter
FLAG-K313 + Reporter
C.
fold enrichment normalized to SFl3
p=0.02
P=0.03
p=0.01
empty vector
FLAG-Wt
FLAG-K313
C/EBPa
PTGS2
IL6Ra
D.
Fold enrichment normalized to SFI3
p=0.532
p=0.052
Wt
K313
C/EBP-LUC
PTGS2
E.
Wild type Peaks
Wild type
K313
Input
-10000 bp
10000 bp
K313 Peaks
K313 cebpa
p42 cebpa
chr1
| 151947000
| 151951000
| 151955000
Ptgs2os
Ptgs2
5162 bp

Fig. S5

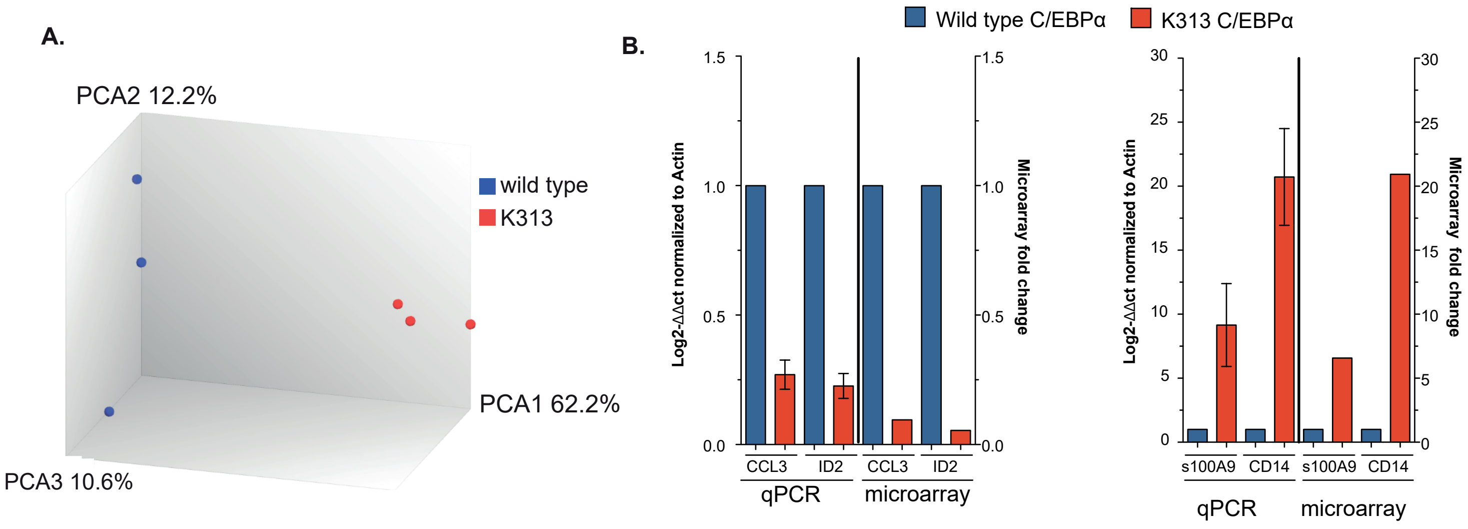
A.
PCA2 12.2%
wild type
K313
PCA1 62.2%
PCA3 10.6%
B.
Wild type C/EBPα
K313 C/EBPα
Log2-ΔΔct normalized to Actin
Microarray fold change
1.5
1.0
0.5
0.0
CCL3
ID2
CCL3
ID2
qPCR
microarray
30
25
20
15
10
5
0
s100A9
CD14
s100A9
CD14
qPCR
microarray

Fig. S6

## Down regulated in cebpa-/- CMP

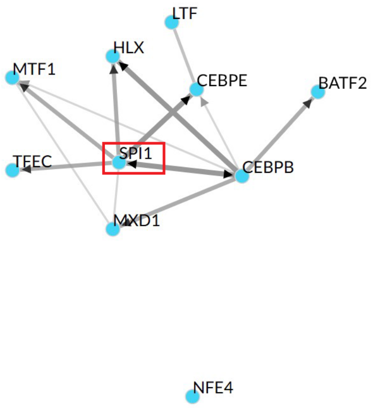


| Rank | TF | Score |
|---|---|---|
| 1 | SPI1 | 5 |
| 2 | TFEC | 6 |
| 3 | CEBPE | 10 |
| 4 | LTF | 16 |
| 5 | MTF1 | 25 |
| 6 | BATF2 | 25 |
| 7 | NFE4 | 27 |
| 8 | HLX | 27 |
| 9 | CEBPB | 28 |
| 10 | MXD1 | 30 |

## Up regulated in K313 cebpa-/- HoxB8

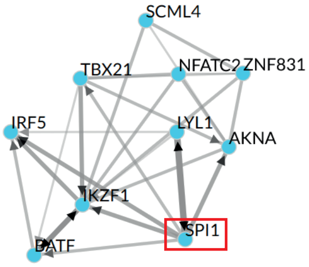


| Rank | TF | Score |
|---|---|---|
| 1 | NFATC2 | 8 |
| 2 | TBX21 | 8 |
| 3 | LYL1 | 16 |
| 4 | SPI1 | 20 |
| 5 | SCML4 | 27 |
| 6 | ZNF831 | 35 |
| 7 | BATF | 37 |
| 8 | IRF5 | 40 |
| 9 | AKNA | 41 |
| 10 | IKZF1 | 41 |

## Up regulated in cebpa-/- CMP

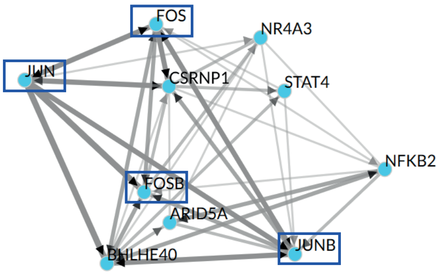


| Rank | TF | Score |
|---|---|---|
| 1 | CSRNP1 | 3 |
| 2 | FOSB | 12 |
| 3 | NR4A3 | 14 |
| 4 | JUNB | 15 |
| 5 | ARID5A | 16 |
| 6 | STAT4 | 19 |
| 7 | BHLHE40 | 22 |
| 8 | FOS | 23 |
| 9 | NFKB2 | 25 |
| 10 | JUN | 27 |

## Down regulated in K313 cebpa HoxB8

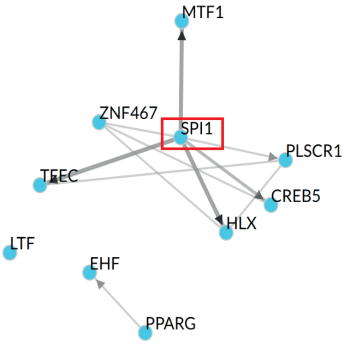


| Rank | TF | Score |
|---|---|---|
| 1 | TFEC | 8 |
| 2 | LTF | 9 |
| 3 | SPI1 | 23 |
| 4 | HLX | 24 |
| 5 | ZNF467 | 32 |
| 6 | CREB5 | 33 |
| 7 | PPARG | 36 |
| 8 | MTF1 | 38 |
| 9 | PLSCR1 | 41 |
| 10 | EHF | 43 |

Figure S7

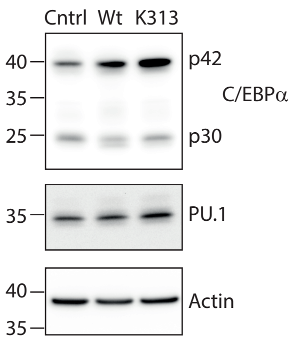


Figure S8

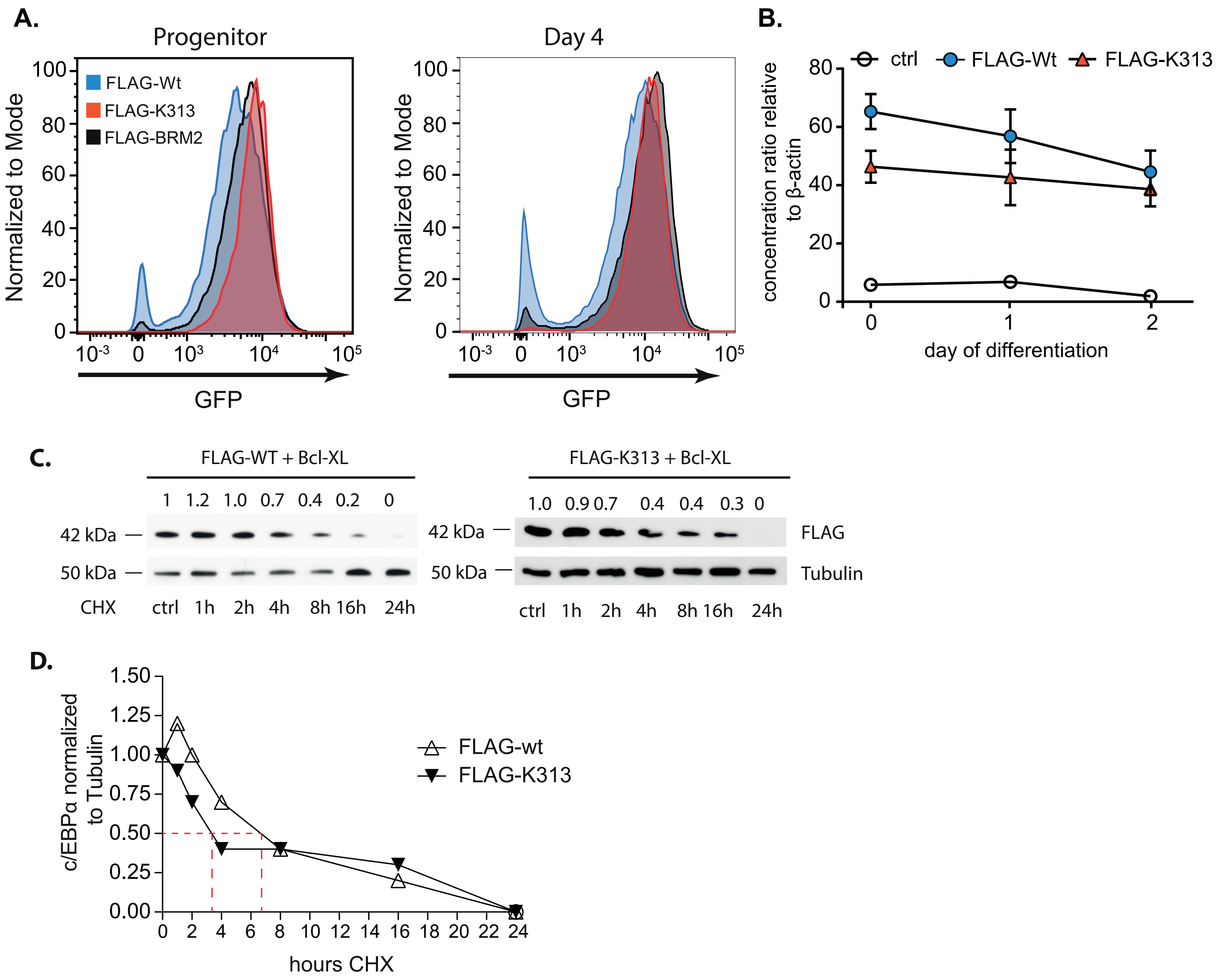
A.
Progenitor
Day 4
FLAG-Wt
FLAG-K313
FLAG-BRM2
Normalized to Mode
100
80
60
40
20
0
10-3
0
10³
10⁴
10⁵
GFP
B.
ctrl
FLAG-Wt
FLAG-K313
concentration ratio relative to β-actin
day of differentiation
C.
FLAG-WT + Bcl-XL
1 1.2 1.0 0.7 0.4 0.2 0
FLAG-K313 + Bcl-XL
1.0 0.9 0.7 0.4 0.4 0.3 0
42 kDa
50 kDa
FLAG
Tubulin
CHX
ctrl 1h 2h 4h 8h 16h 24h
D.
c/EBPα normalized to Tubulin
1.50
1.25
1.00
0.75
0.50
0.25
0.00
0 2 4 6 8 10 12 14 16 18 20 22 24
hours CHX
FLAG-wt
FLAG-K313

Figure S9
